# The Barriers Assessment Tool—A Patient-Centered Measure of Adherence Barriers in Pediatric Kidney Transplantation

**DOI:** 10.3390/children10091435

**Published:** 2023-08-23

**Authors:** Charles D. Varnell, David K. Hooper, Constance A. Mara, Avani C. Modi, Kristin L. Rich

**Affiliations:** 1 Division of Nephrology & Hypertension, Cincinnati Children’s Hospital Medical Center, 3333 Burnet Ave, MLC 7022, Cincinnati, OH 45229, USA; david.hooper@cchmc.org; 2 James M. Anderson Center for Health Systems Excellence, Cincinnati Children’s Hospital Medical Center, Cincinnati, OH 45229, USA; 3 Department of Pediatrics, College of Medicine, University of Cincinnati, Cincinnati, OH 45221, USA; constance.mara@cchmc.org (C.A.M.); avani.modi@cchmc.org (A.C.M.); kristin.loiselle@cchmc.org (K.L.R.); 4 Division of Behavioral and Clinical Psychology, Cincinnati Children’s Hospital Medical Center, Cincinnati, OH 45229, USA

**Keywords:** pediatric kidney transplantation, medication adherence, barriers to adherence, patient-reported outcomes

## Abstract

Objective: Assessing barriers to adherence provides helpful information to clinicians. The objective of this study was to describe the clinical utility of the Barriers Assessment Tool (BAT) using clinical data for a large, midwestern U.S. pediatric kidney transplant program. Methods: Focus group and clinical data were obtained during post-transplant medical visits. Qualitative and quantitative assessment methods were utilized to describe patient and caregiver feedback on the BAT, clinical utility, concordance between reporters, and the effect of interventions on subsequent assessment and electronically measured adherence. Results: Patients were willing to discuss adherence issues with their care team. There was substantial agreement between patients and caregivers at two timepoints. If a barrier was not addressed, 89.6% (43/48) of patients and 85.9% (67/78) of caregivers reported the same BAT scores from the first to second assessment. When barriers were addressed with a clinic-based intervention, 82% of caregivers reported no adherence barriers. No significant change was found for patient-reported barriers. Conclusions: Standardized assessment of barriers to medication adherence provides actionable information to clinicians. Standardized assessment of adherence barriers may give clinicians opportunities to help patients and caregivers overcome these barriers which can decrease risk of rejection.

## 1. Introduction

Suboptimal adherence is a problem across chronic medical conditions, including youth who have received a solid organ transplant. Up to 43% of adolescents with a kidney transplant have suboptimal adherence to their immunosuppression regimen which can lead to an increased risk for acute rejection [1]. Compared to dialysis, a functioning transplant gives increased life expectancy and better quality of life at a lower overall cost [2,3].

Understanding causes of suboptimal adherence through the standardized assessment of barriers is a necessary first step to improving medication adherence. There are several domains that encompass adherence barriers, including cognitive factors, regimen characteristics, lack of knowledge, individual/family factors, financial difficulties, and perceptions about efficacy [4]. Assessment of patient and caregiver adherence barriers reveals that they are common [5,6] and often go unaddressed. In pediatric kidney transplantation, the TAKE-IT randomized clinical trial found significant improvement in adherence for the treatment group (i.e., problem solving around adherence barriers) relative to the control group following an adherence barriers intervention, with both groups endorsing an average of 3–4 adherence barriers at baseline [5]. Unfortunately, evidence-based interventions such as TAKE-IT typically take years to be translated into practice [7,8] and many are never widely implemented at all [9,10,11]. Obstacles to dissemination and implementation of adherence interventions include lack of real-time adherence assessments beyond self-report, difficulty establishing and sustaining reliable clinical pathways, and lack of simple and reliable tools to assess adherence barriers by front-line providers [12].

To address one of the implementation obstacles noted above, we created the Barriers Assessment Tool (BAT), which is a quick and face valid checklist of adherence barriers to be used in a routine kidney transplant clinical practice. The BAT was designed in collaboration with a multidisciplinary kidney transplant team, including a pharmacist, social workers, transplant coordinators, clinic nurses, transplant nephrologists, pediatric psychologists, and patients and caregivers of children and adolescents with a kidney transplant [6]. The BAT was created as a component of the Medication Adherence Promotion System (MAPS) [13] to address suboptimal adherence in clinical practice. Unfortunately, existing validated adherence barrier measures (e.g., Parent and Adolescent Medication Barriers Scales (PMBS and AMBS) [4] and Medication Adherence Measure (MAM) [14]) are lengthy for clinical care and may require a clinical interview format and thus are not feasible for busy clinical outpatient settings. In contrast, the BAT was designed to be completed by the patient and/or caregiver independently and prior to seeing their healthcare provider during clinic.

The goal of this study was to describe the creation and clinical use of the BAT using qualitative and quantitative real-world clinical data. We sought to describe (1) patient and caregiver feedback on barriers to adherence, discussing adherence in clinic, and the BAT; (2) the cost and amount of time it takes for patients and healthcare providers to use the BAT in real-world clinical care to understand its clinical utility; (3) the concordance between a patient and their caregiver on the same date of barriers assessment; (4) the effect of an in-clinic intervention for an adherence barrier on subsequent assessment; (5) the effect of an in-clinic intervention for an adherence barrier on electronically measured adherence. We hypothesized that systematically screening for and helping patients overcome identified barriers in the clinic could improve immunosuppression adherence and decrease acute rejection rates [15], which is a driver of transplant failure.

## 2. Methods

The focus group portion of this study was approved by the Cincinnati Children’s Hospital Medical Center Institutional Review Board (IRB 2019-1025), with a waiver of requirement to obtain documentation of informed consent; however, all patients provided verbal assent prior to focus groups. The remainder of this study was deemed non-human subjects research by the Cincinnati Children’s Hospital Medical Center Institutional Review Board (IRB 2020-0568). The data used were collected for clinical purposes and this study used retrospective data.

### 2.1. Barriers Assessment Tool

The BAT is a 14-item checklist of the most common adherence barriers, with the following broad question “What gets in the way of you taking your immunosuppression medication?” The BAT normalizes that the medication regimen patients are prescribed can be challenging and invites them to share things that get in the way of them taking their medication. Patients and/or their caregivers then select which barriers apply to them. The BAT is in a checklist format (i.e., yes/no) that does not require scoring, compared to measures with Likert-type response options used for research, it is on a single page which makes it quick and easy to complete, and can be easily interpreted and acted upon by clinicians at the point of care. To summarize a patient’s barriers to track changes over time, a simple count of the number of barriers endorsed can be used to create a “score” if needed. However, clinicians may also evaluate each of the barriers endorsed to determine how best to support their patients. The BAT was developed and piloted at Cincinnati Children’s Hospital and has since become part of routine clinical care [6]. It has been adapted to other patient populations [16,17] and is currently being utilized throughout the Improving Renal Outcomes Collaborative [18].

Since 2015, patients in our kidney transplant program have been completing the BAT every six months, or more often if clinically indicated (e.g., increase in psychosocial stressors, recent episode of acute rejection due to nonadherence). The 6-month timeframe was determined based on feedback from patients when the BAT was first developed and used. For patients <10 years old, only the caregiver is asked to complete the BAT. If the patient is ≥10 years old, both patient and caregiver are asked to complete the tool. The age criteria of 10 was chosen because the clinical team believed that pediatric patients could reliably report adherence barriers. If the patient is not able to complete the BAT for any reason (e.g., comprehension, lack of insight, etc.), they can decline to complete the BAT. Patient and caregivers complete the BAT either on a paper form or tablet computer prior to being seen by the clinician, and their responses are entered into a flowsheet within the electronic health record (EHR). The BAT is written at a 5th grade reading level on the Flesch–Kincaid readability scale and exists in English, Spanish, and Arabic. If patients have literacy problems or are cognitively unable to read the measure, the BAT can be read to patients and manually entered into the EHR by staff. All transplant patients are included in screening for the BAT and their data were used for this study. Patients or caregivers were excluded if they declined to complete the BAT.

### 2.2. Procedure

We extracted BAT responses from the EHR from February 2016 to February 2020. Responses were coded to represent whether each patient and/or their caregiver did or did not identify any BAT adherence barriers. All post-transplant patients seen in our outpatient clinic were administered the BAT, unless refused by the patient or caregiver. Each patient was on at least one immunosuppressive medication. If a patient or caregiver identified an adherence barrier and received an adherence intervention in the clinic to help them overcome this barrier, that data were also documented in their EHR. The various clinical interventions have been described elsewhere [13], but briefly, the interventions utilize shared decision making to create an action plan specific to addressing the barrier identified. Descriptive data, including means, standard deviations, and frequencies, were used to characterize participants and adherence and barriers data.

### 2.3. Qualitative Analysis

*Focus Group.* A focus group was conducted to discuss patient and caregiver perceptions of medication adherence, how they want their healthcare teams to discuss medication adherence, and to give feedback on the BAT. The focus group was held at the Improving Renal Outcomes Collaborative (IROC) Fall 2019 Learning Session and consisted of patients and caregivers of patients with a kidney transplant. These patients and caregivers were not involved in the development of the BAT. Verbal consent was obtained to participate in the focus groups and with the collection of questionnaire data and open-ended discussion to understand thoughts and attitudes about immunosuppression medication adherence for themselves or their child. The focus group discussions were recorded and transcribed, with transcriptions analyzed by three separate coders for thematic analysis. Themes identified from patients and caregivers regarding their preferences with discussion adherence issues in the clinic were also noted.

*BAT Clinical Utility*. We assessed the amount of time it takes for a respondent to complete the BAT, the cost of the BAT, and the ease of reviewing patient- or caregiver-reported barriers by the clinician to assess clinical utility. 

### 2.4. Quantitative Analysis

*Patient–Caregiver Agreement.* To complete the quantitative analysis, a BAT “score” was created for each patient at each timepoint, as described above (i.e., a count of the number of barriers endorsed). To assess patient and caregiver agreement, kappa coefficients were calculated to examine convergence between caregivers and patients using the BAT at two separate time points (approximately 6 months apart) (Kappa coefficients: 0 (no agreement), 0.01 to <0.2 (slight agreement), 0.21 to 0.4 (fair agreement), 0.41 to 0.6 (moderate agreement), 0.61 to 0.8 (substantial agreement), >0.81 (almost perfect agreement)) [19].

*Barrier Stability.* To assess barrier stability, we examined barriers for patients/caregivers who reported a barrier to adherence but did not receive a clinical intervention to address the reported barrier, as our hypothesis was that this group would be likely to continue to report the same number of barriers with no intervention. For these stable patients, a McNemar test was conducted for both caregiver and patient-reported barriers from the first-to-second-time BAT completions (approximately 6 months apart).

*Treatment responsiveness.* To assess treatment responsiveness, we assessed two factors: (1) whether patients who received a clinical intervention for a reported adherence barrier still reported the barrier after the clinical intervention (sub-cohort 1) and whether measured adherence changed after a clinical intervention to a reported adherence barrier (sub-cohort 2).

*Changes in Adherence Barriers.* Sub-cohort 1 includes patients and caregivers from the total cohort who completed the BAT before and after reporting an adherence barrier to analyze if patient- and/or caregiver-reported adherence barriers persist after a clinical intervention. McNemar tests were conducted to examine whether barriers were reduced following an adherence intervention.

*Changes in Electronically Monitored Adherence.* Sub-cohort 2 includes patients who participated in a pilot study for patients who were provided with an electronically monitored pillbox (SimpleMed+ by Vaica), an objective adherence measure that collects data in real time. Each time a pillbox compartment was opened, the date and time were uploaded to the Vaica portal. Electronic adherence data were monitored for a baseline period (2–3 months prior to intervention), active treatment (1 month following intervention), and post-treatment (2–3 months following active treatment phase). A chi-square test was used to analyze patients who did or did not identify an adherence barrier based on optimal or suboptimal adherence (suboptimal defined as <95% of prescribed doses taken as prescribed (e.g., for a medication prescribed to be taken twice a day, was it taken twice a day or only once a day?)). There are no data to suggest an optimal rate of adherence in transplant patients but given the significant clinical consequences of less than perfect adherence in transplant patients, we used the cutoff of <95% as suboptimal. Adherence was calculated based on daily electronic monitoring data as follows: ((number of doses taken/number of doses prescribed) × 100). Adherence data were calculated for the 30 days prior to the barriers assessment and associated adherence interventions (baseline) and 30 days following the assessment (post). Due to non-normality of the data, Wilcoxon signed-rank tests were conducted by the group to assess changes in electronically monitored adherence from pre- to post-clinic visit.

## 3. Results

### 3.1. Focus Group

Six individuals (four caregivers, two patients) participated in a focus group on medication adherence. The focus group was conducted at a national conference and following the focus group, participants completed a questionnaire about immunosuppression adherence and the BAT. The focus group questions and results of thematic analysis are displayed in Table 1 and Table 2. From the focus group questionnaire data, half of the respondents indicated that they do not routinely discuss medication adherence with their transplant team, which points to the added utility systematic application of the BAT in clinical practice. Respondents want their transplant teams to know that taking immunosuppression medication is challenging, even under the best circumstances; however, when the patient or family perceives there is a problem, they do feel comfortable discussing this with their transplant team. With regards to the BAT, most respondents indicated that every 3–6 months was an appropriate interval for repeat assessment. Other major themes identified included (1) discussing adherence issues through both direct and indirect approaches; (2) in addition to assessing for barriers, having proactive and solution-based discussions about common barriers to adherence; (3) having frank and honest discussions about the challenges with adherence to immunosuppression protocols, as well as knowing what parts of the regimen are flexible.

### 3.2. BAT Clinical Utility

The BAT takes <60 s for a patient and their caregiver to complete [6]. There is no cost to license and use the BAT and the printing costs are negligible. Due to its checklist format, it is quick and easy to interpret. Anecdotally, healthcare providers who have used the BAT in clinical practice report that it is useful and provides actionable information in the clinic.

### 3.3. Quantitative Analysis

Demographic and medical characteristics for the *n* = 175 patients and caregivers who completed the BAT from February 2016 through February 2020 are summarized in Table 3.

### 3.4. Patient–Caregiver Agreement

Kappa coefficients indicated substantial reliability between caregivers and patients at both time points (Time 1: k = 0.72, *p* < 0.001 (*n* = 79); Time 2: k = 0.74, *p* < 0.001 (*n* = 61).

### 3.5. Barrier Stability

McNemar tests revealed no significant differences in the proportion of endorsed barriers on the BAT for caregivers (McNemar *p* = 0.23) or patients (McNemar *p* = 0.38) for patients and caregiver who did not receive an intervention from Time 1 to Time 2 of BAT completion. Specifically, 89.6% (43/48) of the patients reported the same BAT scores (i.e., same number of barriers) from Time 1 to Time 2, and 85.9% (67/78) of caregivers reported the same BAT scores from Time 1 to Time 2.

### 3.6. Treatment Responsiveness

Changes in adherence barriers. We found that there were 16 unique patients from the cohort of 175 patients (from 14 caregiver reports and 9 patient reports) who completed the BAT before and after an intervention was performed (see Table 3), and thus were included in Sub-cohort 1 to evaluate changes in reported adherence barriers before and after a patient or caregiver received a clinical intervention to address the identified barrier. McNemar tests revealed significant changes for caregiver-reported barriers from Time 1 to Time 2 following receipt of a clinic-based intervention (*p* = 0.02). Specifically, 82% (*n* = 9 of 11) reported no adherence barriers following an intervention compared to 18% (*n* = 2 of 11) who continued to experience adherence barriers following an intervention. No significant change was found for patient-reported barriers (*p* = 0.13).

Electronically monitored adherence. In Sub-cohort 2, 13 kidney transplant patients participated in electronic pillbox monitoring as part of their post-transplant care (see Table 3). Of the 13 participants, more than half identified an adherence barrier at their clinic visit. Forgetting was the most common barrier (71% of those that identified a barrier), with side effects and poor medication taste as additional barriers. One patient identified three total barriers. The use of the shared-decision intervention for forgetting was the most common intervention and most participants chose various reminders (e.g., text messages, cell phone applications) as a method to improve remembering to take their medications.

For the patients who identified barriers, four demonstrated improved adherence, two maintained their adherence, and one demonstrated declining adherence. No significant changes were noted in adherence from pre- to post-treatment for those who endorsed barriers (z = −0.67, *p* = ns); pre-adherence rates = 80.5 ± 10 and post-adherence rates = 85.0 ± 17.7. For the patients who did not identify barriers, adherence either declined (*n* = 2), was unchanged (*n* = 2), or had dramatic improvement (*n* = 1). No significant improvements in adherence were found for those who did not endorse barriers from pre- to post-treatment (z = −0.41, *p* = ns; pre-adherence rate s = 81.5 ± 40 and post-adherence rates = 90.8 ± 7.4). The patients with barriers were more likely to have suboptimal adherence (<95% of doses taken as prescribed) compared to the patients with no barriers identified (*p* < 0.01). Of note, of the participants we approached, 7% refused the electronic monitor due to having an existing system that worked or not wanting to be monitored. An additional 30% experienced technical difficulties with the monitor (e.g., poor cell signals in the patient’s home area).

## 4. Discussion

While there are existing measures of adherence and barriers to adherence [12], our clinical transplant team required a tool to assess barriers that was quick, easy to administer and interpret, and required no training to administer. Additionally, our goal was to begin screening for barriers to adherence without adding new staff to the clinics (e.g., clinical psychologist). The Parent and Adolescent Medication Barriers Scales (PMBS/AMBS) and the Medication Adherence Measure (MAM) are commonly used existing measurement tools in research [4,14]. The PMBS/AMBS covers much of the same material; however, our team wanted a simpler response option and opted for a “yes/no” checklist format where clinicians could easily identify problematic barriers quickly, compared to the construct scores that would be generated from these measures with Likert-type response options. The clinical interview format of the MAM limits its use for the existing staff of a busy transplant clinic.

The results are consistent with past studies assessing barriers and demonstrate that patients are willing to disclose their difficulties to the medical team when given an invitation to do so [4,17]. Depending on the barrier endorsed, patients were able to receive evidence-based interventions in “real time”. When we evaluated the treatment responsiveness for the BAT to identify adherence barriers for patients and caregivers who received a clinical intervention, the caregivers were less likely to report a barrier after receiving a clinical intervention while the patient report did not change. This could be explained by adolescents under or over-reporting perceived adherence barriers, or due to lack of statistical power to detect a significant change. This question would benefit from repeated analysis in a larger cohort of patients.

Data from the electronic adherence monitors revealed that in-clinic intervention had an impact on most of the patients who received it. This shows initial support for the effectiveness of interventions provided by medical providers who do not have extensive behavioral training. Historically, pediatric psychologists have had the unique training to assess and treat barriers and adherence behavior. However, the literature points to greater impact of interventions that are implemented at a system level [20,21]. It is likely that not all barriers can be effectively addressed by the medical team, in which referrals to a behavioral specialist are indicated.

For children, adolescents, and families struggling with their disease management, assessing for adherence barriers is a necessary step in trying to help them overcome these barriers. In pediatric solid organ transplant recipients, Lee and colleagues reported that barriers to medication adherence are stable over time and appear unlikely to change without targeted intervention [22]. Adherence barriers are associated with relevant clinical outcomes, including low drug serum levels, organ rejection, and death [15]. Hunsley and Mash [23] call for assessment tools that are “maximally accurate, efficient, and cost-effective” as well as “brief, clear, clinically feasible, and user-friendly”, the spirit of which is reflected in the BAT. Feedback from the focus group pointed out that it was uncommon for nephrologists to explicitly discuss adherence issues during a routine clinical encounter. Several possible contributing factors may include perceived lack of time, lack of comfort/skill in how to discuss adherence or adherence behavior, and provider’s perception that they know how adherent a patient is based on intuition or other factors; however, this should be further evaluated in future studies. Having the BAT as a piece of clinical data, akin to blood pressure or creatinine levels, may support providers in routinely including a discussion of adherence and adherence barriers into care. Additionally, routine administration of the BAT can promote normalization and facilitate open conversation between providers and families.

This study should be considered in light of several limitations. First, this study includes one kidney transplant clinic in the Midwestern U.S. and may not adequately represent pediatric kidney transplant recipients in other parts of the country or world. Increasing patient diversity in future samples can help determine whether certain barriers are more present for certain demographic groups and how health disparities may impact these. Second, we were limited by small sample sizes in the two sub-cohorts described. Third, there are limitations to electronic pillbox monitoring, including the inability to verify medication ingestion when the box is opened. Additionally, some patients were unable to use the electronic pillbox due to technical issues (e.g., lack of cellular network) or preference (e.g., existing organizational system that is effective). Lastly, to differentiate between optimal and suboptimal adherence in patients who participated with electronic pillbox monitoring, a high cutoff (>95%) was utilized. Future research should explore the range of adherence in kidney transplant recipients that represents risk for poor outcomes.

Overall, the BAT appears to have evidence of validity and reliability and is a promising tool for use in clinical care. Compared to many adherence-related patient-reported outcomes, the BAT has initial support for its use based on the “good enough” Hunsley and Mash criteria [12]. Importantly, the BAT is an example of a tool that was developed with the ease of use and clinical utility in mind. The BAT has been implemented into a busy medical clinic and is providing clinically useful data. It has also been adapted slightly for use with other chronic disease populations, including epilepsy, rheumatological diseases (e.g., lupus, juvenile idiopathic arthritis), cancer, and sickle cell, and can be further adapted for other conditions.

## Figures and Tables

**Table 1:** Focus group interview questions.

Key Questions
Taking medicine for your/your child’s kidney transplant can be difficult.
How is your transplant team currently discussing the importance of taking your/your child’s anti-rejection medication? What would you like your transplant team to review with your regarding how you/your child manage your transplant medicine? How is your healthcare team helpful to you regarding managing medications? What could your healthcare team do to be more helpful regarding managing medications? Transition: Thanks for sharing those thoughts with the group. Now we would like to ask some questions about the Barriers Assessment Tool. Please open your booklet to the Barriers Assessment Tool. This tool was created to ask patients and their family “what gets in the way to taking your anti-rejection medication”. Can anyone share their experience with having used this tool or similar tools like this in clinic? What should be included in this tool that is currently missing or what changes would you make to this tool? What would be the best format to receive and complete this during a clinic visit, for example on a piece of paper, on a tablet computer, etc.

**Table 2:** Thematic analysis of focus groups.

**Questions about Adherence**	**Themes**
1.1: How is your transplant team currently discussing the importance of taking your/your child’s anti-rejection medication?	Direct questions (e.g., any problems taking your medications?).
	Indirect questions (e.g., do you need medication refills, do you know why you take your medications?).
1.2: What would you like your transplant team to review with you regarding how you/your child manages your transplant medication?	Proactive and preventative discussion about barriers to adherence (e.g., counseling on common barriers that patients experience).
	Discussions that emphasize barriers are to be expected and not a patient or caregiver failure.
	Use open-ended, non-judgmental approach to discussions.
	Use solutions-based, age-appropriate conversation.
	Talk directly to the patient—even if child.
1.3: How is your healthcare team helpful to you regarding managing medications?	Direct questioning about what medications the patient takes it, how often, why.
1.4: What could your healthcare team do to be more helpful regarding managing medications?	Transplant team assumes what we know and do not know. The parents would rather be told repeated information that already know than miss something the team assumes they know with regards to medication.
	Normalizing the experience of having a kidney transplant and taking medication.
	Discuss flexibility that may exist within a treatment plan.
**Questions about Barriers Assessment Tool**	**Themes**
2.1: Can anyone share their experience with having used this tool or similar tools like this in clinic?	N/A: none of the participants have used this or a similar tool in clinic before.
2.2: What should be included in this tool that is currently missing or what changes would you make to this tool?	Include barriers around insurance issues that can affect obtaining medication.
	Consider creating a logic so if a patient endorses a barrier there are follow-up questions.
	Rephrase the questions for activities such that “my other activities get in the way of taking my medication”.
2.3 What would be the best format to receive and complete this during a clinic visit, for example on a piece of paper, on a tablet computer, etc.	Participants agreed having both paper versions and electronic options are important for different types of patients and families.
	Version where BAT is able to be pushed out to parents ahead of visit to be carried out at home.

**Table 3:** Demographic data for patients who completed the BAT.

Patient Characteristic ^a^	Total Cohort (*n* = 175)	Sub-Cohort 1 (*n* = 16)	Sub-Cohort 2 (*n* = 13)
**Age in years (Mean (SD))** **Patients < 10 years old**	12.8 (6.6) 65 (37%)	12.6 (5.0) 7 (44%)	16.8 (4.7) 0
**Sex**			
Male	95	10	9
Female	80	6	4
**Race**			
Asian	7	0	0
Black	25	2	6
Hispanic/Latino	5	0	0
Middle Eastern	1	0	0
White	137	14	7
**End-Stage Kidney Disease Cause**			
CAKUT	73	6	4
Genetic	37	1	4
Glomerular	35	5	2
Immune mediated	3	0	0
Medication	5	0	0
Oncologic	2	1	0
Other	13	3	1
Unknown	2	0	1
Vascular	5	0	1
**Type of transplant**			
Living	109	10	7
Deceased	66	6	6
**Transplant episode**			
First	165	14	13
Second	9	2	0
Third	1	0	0
**Immunosuppression ^b^**			
Tacrolimus	91	8	8
Sirolimus	14	2	5
Cyclosporine	5	1	0
Mycophenolate	72	8	8
Azathioprine	30	1	4
Leflunomide	1	0	1
Steroids	36	6	5
Belatacept	1	0	0
**Years since transplant**	3.1	2.5	5.9

^a^ Time-dependent variables (i.e., age, transplant episode, primary IS, years since transplant are calculated using the date of first completion of the BAT). ^b^ Most patients are on more than one immunosuppression medication, so total will be greater than *n*.

## Data Availability

The data presented in this study are available upon reasonable request from the corresponding author. The data are not publicly available due to privacy concerns.
